# Role of oxidative/nitrosative stress in dysfunction of rat’s intracerebral parenchymal arterioles in low sodium environment in the presence of vasopressin

**DOI:** 10.1007/s00424-025-03097-1

**Published:** 2025-05-28

**Authors:** Marta Aleksandrowicz

**Affiliations:** Laboratory of Preclinical Research and Environmental Agents, Mossakowski Medical Research Institute, Polish Academy of Sciences, A. Pawińskiego Str. 5, 02-106 Warsaw, Poland

**Keywords:** Cerebrovascular dysfunction, Hyponatremia, Parenchymal arterioles, Peroxynitrite, ROS, Vasopressin

## Abstract

Hyponatremia is the most common electrolyte disturbance in hospitalized patients. Symptoms of hyponatremia include attention deficits and cognitive impairments. The cause of such abnormalities may be disturbances in the regulation of microcirculation. Previous studies have shown that increased vasopressin (AVP) concentration to 15 pg/ml in the presence of decreased Na^+^ concentration to 121 mM, which mimics AVP-associated hyponatremia in vivo leads to dysfunction, i.e., constriction and impaired endothelial regulation of small intracerebral blood vessels—parenchymal arterioles (PA). One of the possible causes of this dysfunction may be excessive production of superoxide anion (O2^•−^). The superoxide anion binds nitric oxide (NO) in a reaction that produces aggressive nitrogen-free radical, peroxynitrite (ONOO^−^), which simultaneously reduces the bioavailability of NO. The present studies were performed in the organ chamber on isolated, perfused, and pressurized rats’ PA in low sodium environment in the presence of AVP. These studies aimed to investigate the mechanism leading to PA dysfunction, i.e., constriction and disturbed endothelial regulation. L-NAME (N(ω)-nitro-L-arginine methyl ester) did not elicit constriction of PA, indicating reduced involvement of NO in maintaining basal tone of PA. Vasopressin V_1a_ receptor antagonist (SR 49059), endothelin ET_A_/ET_B_ receptors antagonist (PD 142,893), peroxynitrite decomposition catalyst (FeTMPyP) and ROS scavengers: superoxide dismutase (SOD) and catalase (CAT) improved studied responses. Dihydroethidium (DHE) staining confirmed the increased superoxide anion formation in low sodium environment in the presence of AVP. Thromboxane A_2_/prostaglandin H_2_ receptor blocker (SQ 29,548), an inhibitor of the production of 20-HETE (HET0016), and L-arginine, a precursor of NO, did not improve dysfunctions of PA. Thus, in studied conditions, endothelial dysfunction occurs due to oxidative/nitrosative stress. These findings provide novel insight into the detrimental effects of decreased Na^+^ concentration in the presence of increased AVP concentration that mimic hyponatremia, on the regulation of cerebral microcirculation.

## Introduction

Hyponatremia, defined as a decrease in the plasma sodium concentration below 135 mM, is the most common electrolyte abnormality reported in hospitalized patients [7]. Except in cases of water intoxication, hyponatremia usually occurs in the presence of increased plasma levels of vasopressin (AVP) [7]. This water-electrolyte balance disorder often accompanies neurological diseases such as subarachnoid hemorrhage, brain tumor, and head trauma and is considered a poor predictor for these patients [26, 40]. The contribution of hyponatremia alone to neurologic manifestations and the underlying mechanisms remain unclear, mainly because underlying diseases may also affect brain function [26]. Multiple sources report that the adverse effects of hyponatremia on the patients, as well as its symptoms, such as headache, confusion, and attention deficits, might be related to hypoosmotic cerebral edema [7, 24, 40, 42]. Especially, interstitial edema and perivascular astrocyte foot process swelling are present [36], while neuronal elements are spared [6, 36]. Additionally, in response to cerebral edema, increased intracranial pressure may lead to a decline in cerebral blood flow and cerebral herniation with hypoxia [5]. Another possible cause of neurological deficits may be a disturbed metabolic regulation of the cerebral microcirculation. Blood flow to this part of the cerebral circulation depends on the function of the small intracerebral vessels—parenchymal arterioles (PAs). Previous studies showed that the increased plasma concentration of vasopressin (AVP) in the presence of decreased Na^+^ concentration leads to dysfunction, i.e., vasoconstriction and impaired parenchymal arterioles tone regulation. Among other observations, the impaired response of PA to adenosine triphosphate (ATP) was observed [2, 3], indicating a dysfunction of the endothelium [35]. Brain endothelial cells are part of the neurovascular unit (NVU)—the structural and functional element of the brain responsible for delivering blood to the microcirculation in an amount sufficient to cover the current metabolic demand. This process of functional hyperemia is also known as neurovascular coupling (NVC) [39]. Parenchymal arterioles participate in this hyperemic response, and impaired function of these vessels is considered the most frequent cause of vascular cognitive impairment [22]. Attention deficits, cognitive impairment, and gait disturbances were reported both in experimental models [19, 30] and in patients with hyponatremia [23].

Endothelial dysfunction is commonly caused by the oxidative stress associated with the increased production of oxygen free radicals [15]. One of them, superoxide anion (O2^•−^), reacts with NO to produce peroxynitrite, an aggressive nitrogen-based free radical, which disrupts endothelial caveolae leading to the endothelial nitric oxide synthase (eNOS) uncoupling and reduced NO bioavailability. Uncoupled eNOS produces superoxide anion (O2^•−^) instead of NO, creating a vicious circle [11]. Dysfunctional endothelium secretes vasoconstrictive endothelin-1 (ET-1) and thromboxane A_2_ (TXA_2_) instead of vasodilators such as NO and prostacyclin (PGI_2_) [15, 20, 24]. Endothelial NOS uncoupling and reduced NO bioavailability have other implications for cerebral blood vessels. It is known that part of the vasodilatory action of NO in the cerebral circulation is caused by the direct inhibition of the production of 20-hydroxyeicosatetraenoic acid (20-HETE) in smooth muscle cells [28]. 20-HETE, a product of omega-hydroxylation of arachidonic acid, is a potent vasoconstrictor. When NO is not available, the production of 20-HETE is not inhibited, and its vasoconstrictor effects are unmasked.

The present studies aim to investigate the mechanisms involved in the dysfunction, i.e., constriction and disturbed endothelial regulation of the isolated parenchymal arterioles in low sodium environment in the presence of vasopressin, with particular emphasis on the contribution of NO in maintaining basal tone of PA and role of V_1a_ receptor, 20-HETE, peroxynitrite, oxygen free radicals, endothelin-1, thromboxane A_2_ and deficiency of NO precursor – L-arginine in this dysfunction.

## Material and methods

### Animals

Male Wistar rats (body weight 250–300 g, *n* = 52) used in this study were supplied by the Animal House of Mossakowski Medical Research Institute, Warsaw, Poland. All experiments were performed ex vivo, in accordance with the Law and Regulations on Animal Protection in Poland (Dz. U 2015/266).

### Preparation of cerebral vessels and pressurized arteriograph

**Fig. 1:**
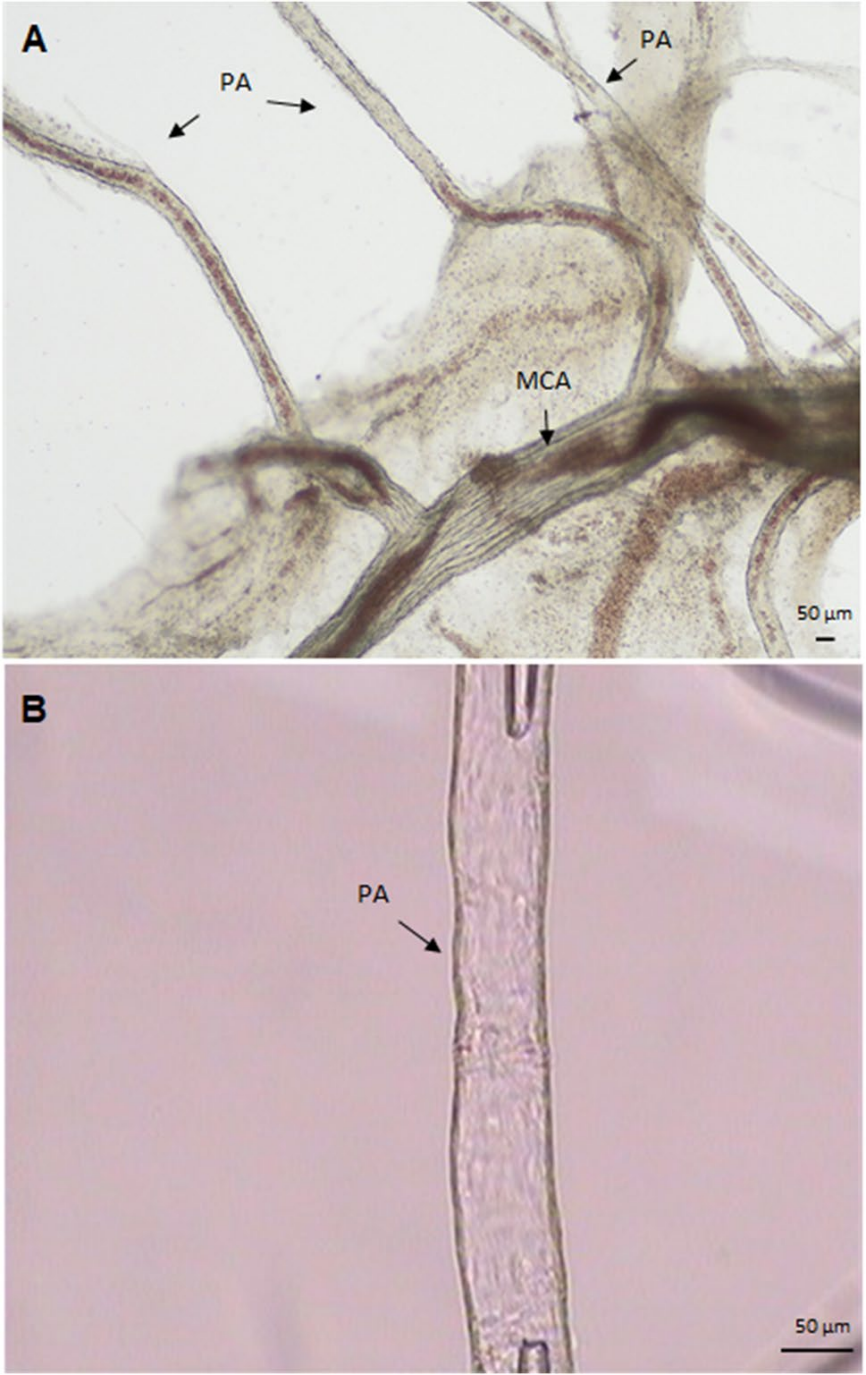
**A** Image of a rat’s isolated parenchymal arterioles (PAs) identified as the intraparenchymal middle cerebral artery (MCA) branches, **B** Image of a cannulated, sutured, and pressurized rat PA. Bar = 50 µm

The rats were anesthetized with 5% isoflurane in 70% NO2/30% O_2_ and decapitated. The brain was placed in a cold (4 °C) physiological saline buffered with MOPS (3-morpholinopropane sulphonic acid) (MOPS-PSS) with the addition of 1% dialyzed bovine serum albumin (BSA). MOPS-PSS was composed of (in mM): MOPS 3.0; NaCl 144.0; KCl 3.0; CaCl_2_ 2.5; MgSO_4_1.5; NaH_2_PO_4_1.21; EDTA 0.02; pyruvate 2.0; glucose 5.0. The parenchymal arterioles, identified as the intraparenchymal middle cerebral artery branches (Fig. 1A), were cut out from the brain parenchyma under a stereoscopic microscope (SZX16, Olympus, Germany). The vessels were transferred to the arteriograph chambers (custom-made pressure-flow chamber and Living Systems Instrumentation, Burlington, VE, USA), cannulated with glass micropipettes containing PSS with 1% BSA and secured with fiber pulled from silk threads (Fig. 1B). Two vessels were studied simultaneously. The arteriograph chambers were placed on the stage of an inverted microscope (CKX53/CKX 41, Olympus, Germany) equipped with a video camera and a monitor. The vessels were perfused at physiological pressure (60 mmHg) with a standard MOPS-PSS buffer containing 1% BSA. The vessels were stabilized for 1 h at 37 °C to develop spontaneous myogenic tone. Subsequently, the vessels were subjected to a viability test by measuring their response to the increase in KCl concentration in the extravascular buffer from 3 to 10 mM (leading to hyperpolarization and dilation) and to 60 mM (leading to depolarization and maximal constriction). Next, the buffer was replaced with the standard MOPS-PSS. Vessels that failed KCl tests were discarded. A measured variable was the internal diameter of the vessel.

### Isolated vessels experimental protocol

All experiments were performed in vitro in the organ chamber. First, AVP was added to the chamber filled with standard MOPS-PSS (144 mM Na^+^, osmolality 300 ± 2 mOsm/kg H_2_O) at a concentration of 15 pg/mL. After 30 min of perfusion with normal sodium and AVP, the Na^+^ concentration in the intra- and extravascular buffer was decreased to 121 mM for 1 h without a change in the concentration of AVP. The osmolality of the low sodium buffer was 260 ± 5 mOsm/kg H_2_O. The concentrations of AVP and Na^+^ were similar to those observed in the plasma of neurosurgical patients with a syndrome of inappropriate antidiuretic hormone secretion [10, 25, 33].

To determine the effect of NO synthase inhibition on the resting tone of the PA, L-NAME (N(ω)-nitro-L-arginine methyl ester, 100 µM) was administered 1 h after the changing the buffer to the one containing a reduced concentration of sodium ions and an increased concentration of AVP. Change in diameter was measured 30 min later.

To assess the effect of osmolality on constriction and abolished response of parenchymal arterioles to ATP in low sodium environment in the presence of vasopressin, the osmolality of the buffer was maintained at 300 ± 2 mOsm/kg with N-methyl-D-glucamine (NMDG).

To determine the role of vasopressin V_1a_ receptor, endothelin ET_A_/ET_B_ receptors, thromboxane A_2_ receptor, 20-HETE, peroxynitrite, and ROS formation on the PA constrictions, the vasopressin V_1a_ receptor antagonist (SR 49059, 100 nM), endothelin ET_A_/ET_B_ receptors antagonist (PD142,893, 100 nM), thromboxane A_2_/prostaglandin H_2_ (PGH_2_) receptor antagonist (SQ 29,548, 2 µM), an inhibitor of 20-HETE (HET0016, 10 µM) production, peroxynitrite decomposition catalyst (FeTMPyP, 10 µM) and ROS scavengers: superoxide dismutase (SOD; 200 U/mL) and catalase (CAT, 150 U/mL) were added extraluminally to the physiological buffer before switching to a buffer that contained low sodium and vasopressin. For the first 30 min, only the test substances circulated in the buffer containing 144 mM Na^+^; then, for the next 30 min, the buffer was changed to one containing the test substances and vasopressin. After this stage, all tested substances (SR 49059, PD142,893, SQ 29,548, HET0016, FeTMPyP, SOD/CAT) were added to the buffer with decreased sodium ion and increased AVP concentration to assess if they influenced PA constriction. Since the reduction of sodium ion concentration in the intra- and extravascular buffer alone (without the addition of vasopressin) also induced a slight constriction of parenchymal arterioles [2, 3], we investigated whether SR 49059, PD142,893, SQ 29,548, HET0016, FeTMPyP, and SOD/CAT would affect this constriction. For the first 30 min, only the test substances circulated in the buffer containing 144 mM Na^+^; then, for the next 1 h, the buffer was changed to one containing the test substances in the buffer with decreased sodium ion concentration.

To determine whether ROS formation, peroxynitrite, and deficiency of NO precursor, L-arginine, are involved in the abolished response of the parenchymal arterioles to ATP, ROS scavengers superoxide dismutase (SOD; 200 U/mL) and catalase (CAT, 150 U/mL), peroxynitrite decomposition catalyst FeTMPyP (10 µM) and L-arginine (1 nM) were present in the buffer during the studying of the responses of the parenchymal arterioles to ATP (100 pM–10 nM). The diameter of the vessel in response to each dose of ATP was measured 15 min after its administration. At the end of these experiments, 1 mM EGTA was administered. Experiments with FeTMPyP were performed in the dark.

### Detection of superoxide formation

The formation of superoxide anion was assessed in the isolated PAs by the dihydroethidium (DHE) fluorescence. DHE is oxidized by O2^•−^ to ethidium bromide, which subsequently intercalates with DNA and is trapped in cell nuclei [45]. The PAs were isolated and located in the organ chamber containing MOPS-PSS. Next, the buffer was changed to one containing low sodium (121 mM) and vasopressin (15 pg/ml). The control group consisted of the vessels incubated in physiological conditions. After 1 h, the MOPS-PSS buffer was changed to buffered phosphate saline (PBS). Hoechst (1:2,000) was administered to the chamber for 5 min to stain the nuclei. The vessels were washed out with PBS and incubated with dihydroethidium (10 mM) at 37 °C for 25 min in the dark. The vessels were rinsed with PBS for 5 min, mounted with VECTASHIELD Vibrance Antifade Mounting Medium, and coverslipped. Fluorescence images of arterioles from the control and low sodium/AVP group were visualized using a confocal laser scanning microscope (LSM 780, Carl Zeiss, Germany) with an × 20/0.8 plan-apochromat objective and Zen black software. Ethidium fluorescence and nuclei Hoechst were excited at 560 nm and 405 nm, respectively. Nuclei were identified according to their Hoechst signal, and from these regions, the ethidium fluorescence was quantified. The images were performed on the day of the experiment and analyzed using ImageJ software.

### Reagents

Buffer was prepared fresh before each experiment. All used reagents were purchased from Merck, except SQ 29,548, which was purchased from Cayman Chemical, DHE (Invitrogen by Thermo Fisher Scientific), and VECTASHIELD Vibrance Antifade Mounting Medium (VectorLabs). SR 49059, HET0016, DHE, and SQ 29,548 were dissolved in DMSO. The final concentration of DMSO in the MOPS buffered saline did not exceed 0.025%. SOD and CAT were dissolved, respectively, in 10 mM and 50 mM KH_2_PO_4_. The remaining reagents were dissolved in distilled water. CAT was not frozen after pipetting.

### Data calculations and statistical analysis

Changes in the diameter of the PAs in response to the compounds involved in the study of the constriction mechanism and to L-NAME were calculated using the equation: (D_active_/D_baseline_) × 100%, where D_active_ is the diameter after drug administration and D_baseline_ is the diameter prior to drug administration.

The percentage dilation of PAs in response to compounds involved in study of disturbed response to ATP was calculated from the equation: (D_active_ − D_baseline_)/(D_maximum_ – D_baseline_) × 100%, where D_active_ is the diameter obtained after drug administration, D_baseline_ is the diameter prior to drug administration, and D_maximum_ is the diameter in calcium-free MOPS-PSS with 1 mM EGTA.

Statistical analyses were performed using Statistica software. Results are expressed as means ± S.E.M. Differences between the groups were analyzed using paired or unpaired Student’s t-test. Differences with *p* < 0.05 were considered statistically significant.

## Results

**Fig. 2:**
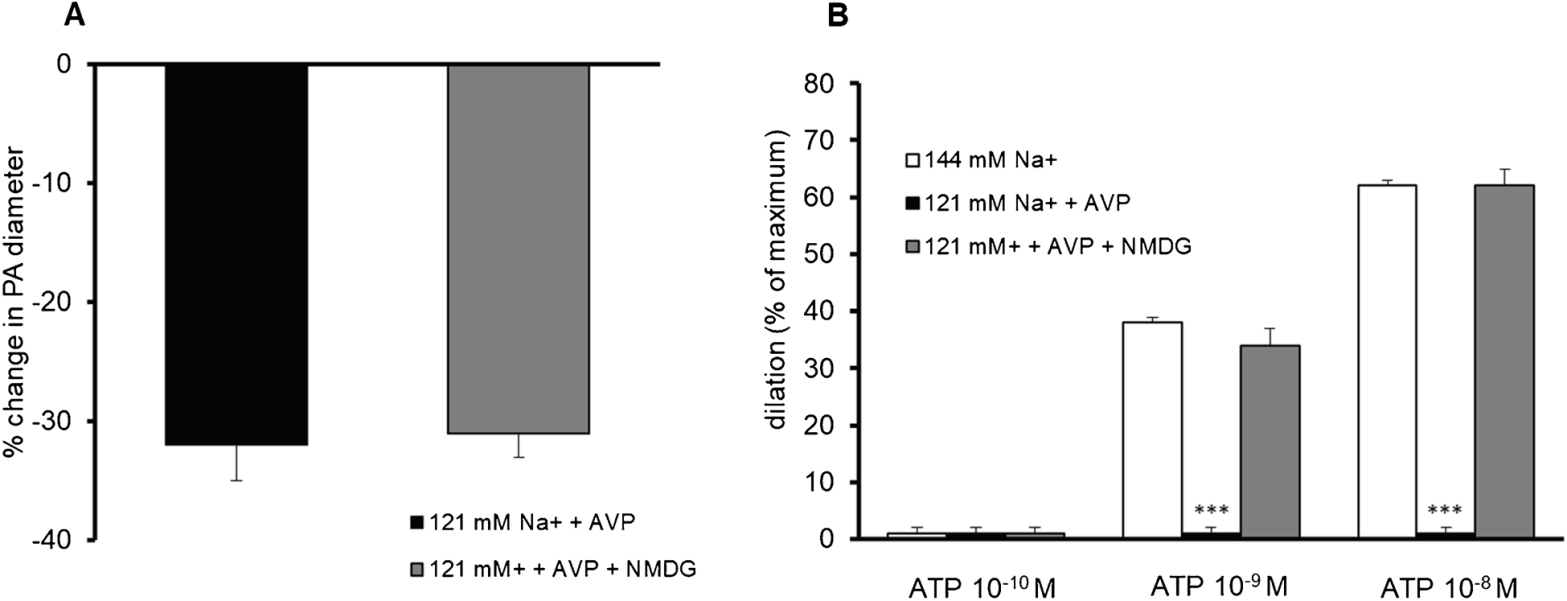
The effect of the correction of osmolality to 300 ± 2 mOsm/kg with N-methyl D-glucamine (NMDG) on constriction (*n* = 9) and the disturbed response of parenchymal arterioles to ATP (100 pM – 10 nM, *n* = 5) in low sodium environment (121 mM Na^+^) in the presence of vasopressin (15 pg/ml). The correction of osmolality with NMDG did not affect constriction of parenchymal arterioles, *n* = 5 (**A**) but restored the response of parenchymal arterioles to ATP, *n* = 5 (**B**). Data are expressed as means ± S.E.M., ***p < 0.001 vs. physiological conditions (i.e., 144 mM Na.^+^ concentration)

Parenchymal arterioles, in response to increased intravascular pressure to 60 mmHg, constricted from 66 ± 3 µm to 39 ± 2 µm (40 ± 1%, *p* < 0.001, *n* = 41). AVP added to the physiological buffer caused relaxation of the PA by 27 ± 4% (*p* < 0.05, *n* = 11). Changing the physiological buffer to that containing a decreased concentration of Na^+^ to 121 mM and an increased concentration of AVP to 15 pg/ml caused constriction of PA by 31 ± 5% (*p* < 0.001, *n* = 9). In such conditions, the response of parenchymal arterioles to ATP (10^−10^–10^−8^ M) was completely abolished compared with physiological conditions, in which dilation started from the threshold concentration of 1 nM [2]. Constriction of PA observed in the low sodium environment in the presence of vasopressin was not affected by adjustment of osmolarity with NMDG to physiological levels. However, osmolarity correction restored the response of parenchymal arterioles to ATP (10^−10^–10^−8^ M, *n* = 5) (Fig. 2).

### Effect of L-NAME on PA diameter in physiological conditions and in low sodium environment in the presence of vasopressin

**Fig. 3:**
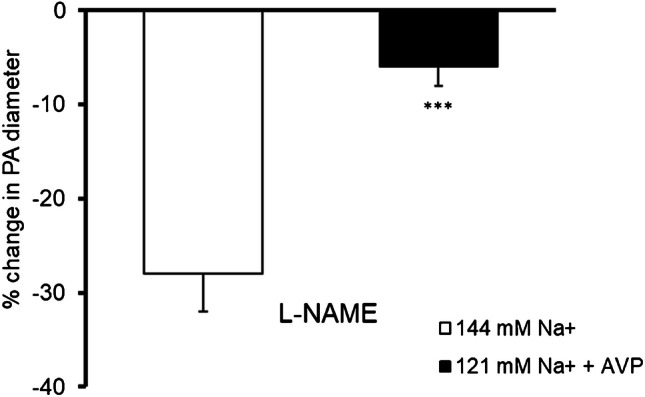
Effect of NO synthase inhibitor, L-NAME (N(ω)-nitro-L-arginine methyl ester, 100 µM), on the PA diameter. In physiological conditions (i.e., normal Na^+^ concentration, *n* = 5), L-NAME led to constriction of PA. In contrast, in low sodium environment (121 mM Na^+^) in the presence of vasopressin (15 pg/ml) (*n* = 6), this response was abolished, indicating decreased involvement of NO in maintaining basal tone. The change in PA diameter was calculated as a percentage of the baseline diameter. Data are expressed as means ± S.E.M., ***p < 0.001 means the difference in response of PA to L-NAME between physiological conditions (i.e., 144 mM Na^+^ concentration) and 121 mM Na^+^/15 pg/ml AVP conditions

Cerebral arterioles have basal NO production [12, 16]. L-NAME, an NO synthase inhibitor, increases vascular tone by eliminating basal NO production [10]. In present studies in physiological conditions, extravascular administration of L-NAME (100 µM) evoked constriction of PA by 28 ± 4% (*p* < 0.05, *n* = 5). In contrast, in low sodium environment in the presence of vasopressin, L-NAME did not influence the vessel diameter (*n* = 6), i.e., vasoconstriction was completely abolished (Fig. 3).

### Effect of SR 49059, PD142,893, SQ 29,548, HET0016, FeTMPyP, and SOD/CAT on parenchymal arteriole basal tone and vasopressin-induced relaxation in physiological conditions

**Fig. 4:**
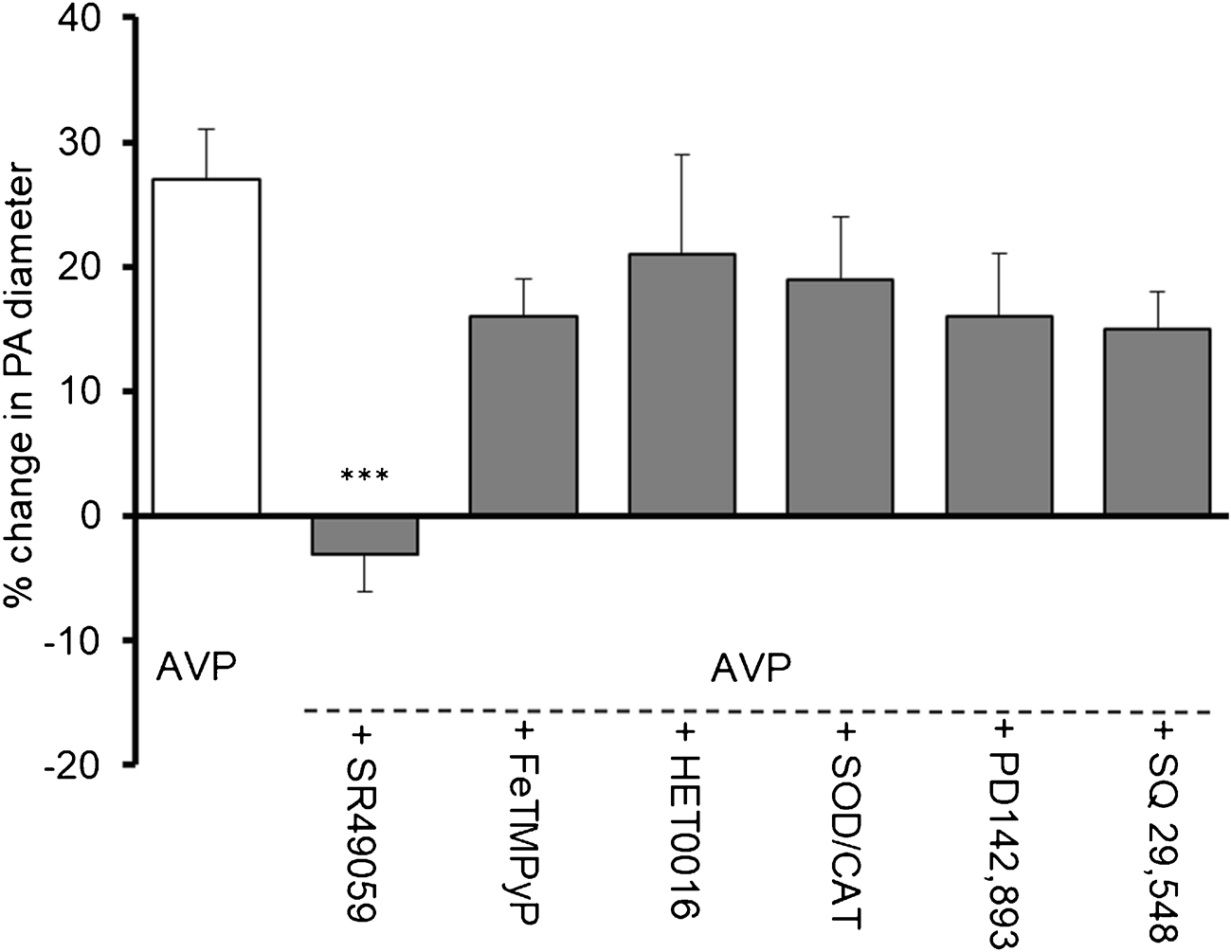
The effect of vasopressin V_1a_ receptor antagonist SR 49059 (100 nM, *n* = 5), peroxynitrite decomposition catalyst (FeTMPyP, 10 µM, *n* = 5), inhibitor of synthesis of 20-hydroxyeicosatetraenoic acid (HET0016, 10 µM, *n* = 5), ROS scavengers: superoxide dismutase (SOD; 200 U/mL) and catalase (CAT, 150 U/mL, *n* = 5), endothelin ET_A_/ET_B_ receptors antagonist (PD142,893, 100 nM, *n* = 5) and thromboxane A_2_/prostaglandin H_2_ (PGH_2_) receptor blocker (SQ 29,548, 2 µM, *n* = 4) (gray bars) on relaxation of parenchymal arterioles to AVP (15 pg/ml, *n* = 11, white bar). Experiments were performed in physiological conditions (i.e., 144 mM Na^+^ concentration). Data are expressed as means ± S.E.M., ***p < 0.001 vs. response of parenchymal arterioles to AVP

Before the concentration of sodium ions in the intra- and extravascular fluid was reduced, the influence of agents involved in the study of vascular responses on the basal tone of parenchymal arterioles was examined under physiological conditions, i.e., with a normal concentration of sodium ions (Table 1). Then, under physiological conditions, the influence of these substances on the vasodilatory action of vasopressin (15 pg/ml) was studied. Among the tested substances, the vasopressin V_1a_ receptor antagonist, SR 49059 completely abolished the response of parenchymal arterioles to vasopressin (Fig. 4).

**Table 1:** The effect of reagents involved in the study of vascular responses on the diameter of parenchymal arterioles under physiological conditions, i.e., with normal sodium ion concentration in intra- and extravascular fluid

Reagent	Dose	Effect on PA diameter
SR 49059	100 nM	No change, 5 ± 3% (NS, *n* = 5)
PD142,893	100 nM	Dilation by 16 ± 4% (*p* < 0.01, *n* = 10)
SQ 29,548	2 µM	Dilation by 12 ± 3% (*p* < 0.01, *n* = 9)
HET0016	10 µM	Dilation by 8 ± 3% (*p* < 0.05, *n* = 11)
FeTMPyP	10 µM	Constriction by 9 ± 3% (*p* < 0.05, *n* = 10)
SOD/CAT	200 U/mL/150 U/ml	No change 6 ± 2% (*p* < NS, *n* = 9)

### Mechanism of constriction of parenchymal arterioles in low sodium environment in the presence of vasopressin

**Fig. 5:**
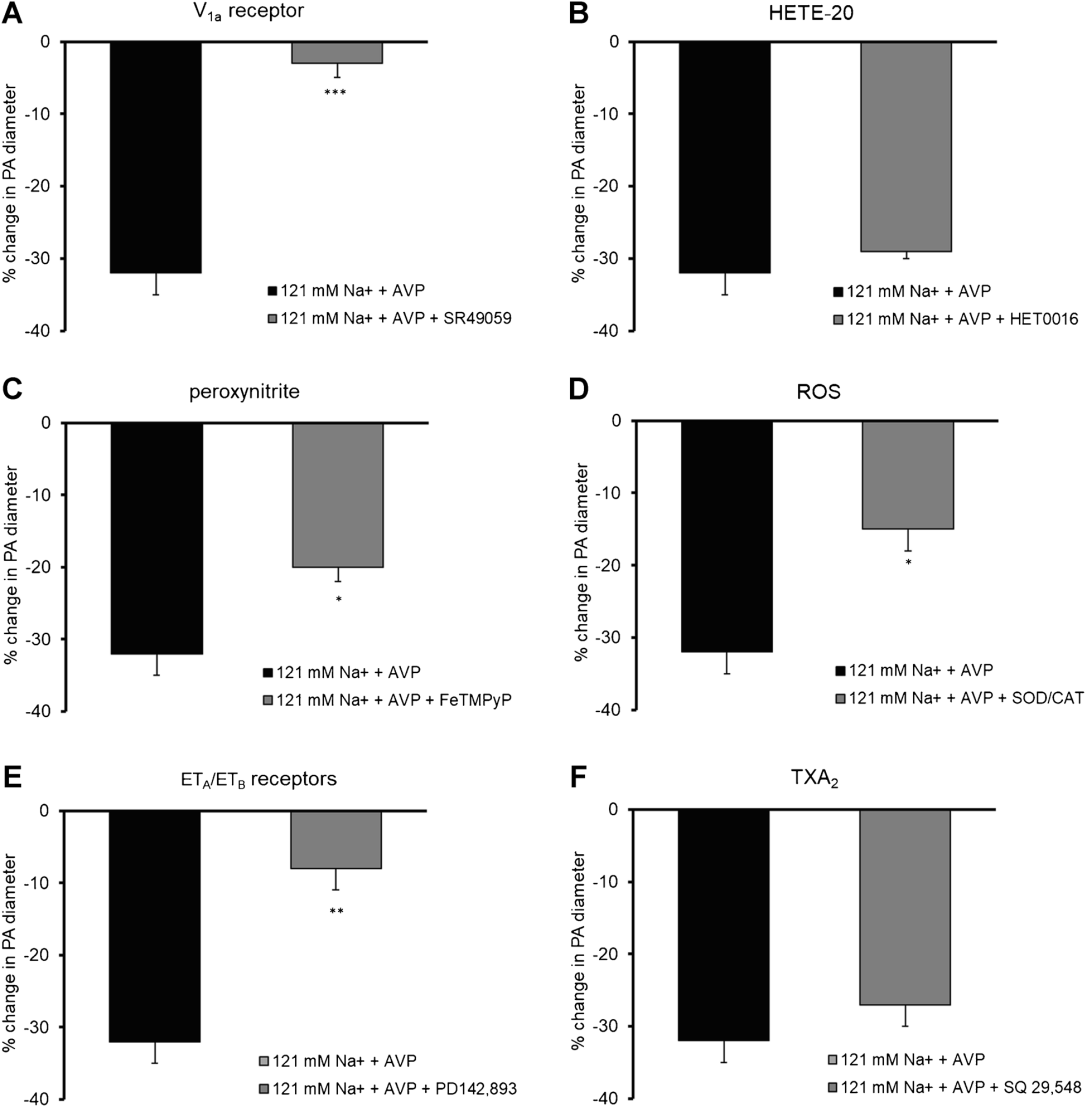
Mechanisms of constriction of parenchymal arterioles in low sodium environment (121 mM Na^+^) in the presence of vasopressin (15 pg/ml). **A** The vasopressin V_1a_ receptor antagonist SR 49059 (100 nM, *n* = 5) abolished constriction of PA; **B** HET0016 (inhibitor of synthesis of 20-hydroxyeicosatetraenoic acid, 20-HETE, 10 µM, *n* = 5) did not influence constriction of PA; **C** Peroxynitrite decomposition catalyst (FeTMPyP, 10 µM, *n* = 5) diminished constriction of PA; **D** ROS scavengers: superoxide dismutase (SOD; 200 U/mL) and catalase (CAT, 150 U/mL, *n* = 5) diminished constriction of PA; **E** Endothelin ET_A_/ET_B_ receptors antagonist (PD142,893, 100 nM, *n* = 5) abolished constriction of PA; **F** Thromboxane A_2_/prostaglandin H_2_ (PGH_2_) receptor blocker (SQ 29,548, 2 µM, *n* = 4) did not influence constriction of PA observed in low sodium environment in the presence of vasopressin. The change in PA diameter was calculated as a percentage of the baseline diameter. Data are expressed as means ± S.E.M., *p < 0.05, **p < 0.01, ***p < 0.001 vs. 121 mM Na^+^/15 pg/ml AVP conditions

Since AVP (15 pg/ml) was used in the present studies, it was first assessed if vasoconstriction of PA was associated with activation of the vascular V_1a_ receptor. Administration of SR 49059 (100 nM, *n* = 5) completely abolished the constriction of PA in conditions of decreased Na^+^ concentration in the presence of increased AVP concentration (Fig. 5A).

NO deficiency may unmask the vasoconstrictive effect of 20-HETE [28]. Incubation of the vessels with a selective inhibitor of the biosynthesis of 20-HETE, HET0016 (10 µM, *n* = 5), caused PA constriction by 29 ± 1%, indicating that HET0016 did not influence the vasospasm observed in low sodium environment in the presence of vasopressin (32 ± 3%) (Fig. 5B).

Enhanced basal tone is often associated with oxidative/nitrosative stress [15, 18, 37]. Pretreatment of the vessels with peroxynitrite decomposition catalyst (FeTMPyP, 10 µM, *n* = 5) and ROS scavengers: superoxide dismutase (SOD; 200 U/mL) and catalase (CAT, 150 U/mL, *n* = 5), significantly diminished constriction of PA to 16 ± 2% (*n* = 5, *p* < 0.001) and 15 ± 3% (*n* = 5, *p* < 0.001), respectively. These results demonstrated the significant role of nitrosative and oxidative stress in constriction of parenchymal arterioles observed in low sodium environment in the presence of vasopressin (Fig. 5C, D).

Since dysfunctional endothelium may release excess vasoconstrictive substances such as endothelin-1 and thromboxane A_2_ [14, 15], their participation in the constriction of parenchymal arterioles was assessed. The presence of endothelin ET_A_/ET_B_ receptors antagonist PD142,893 (100 nM, *n* = 5) in low sodium/AVP buffer abolished constriction of vessels (8 ± 3% vs. 32 ± 3%, *p* < 0.01) (Fig. 5E). In contrast, the thromboxane A_2_ receptor antagonist (SQ 29,548, 2 µM, *n* = 4) did not influence vasoconstriction (Fig. 5F).

**Fig. 6:**
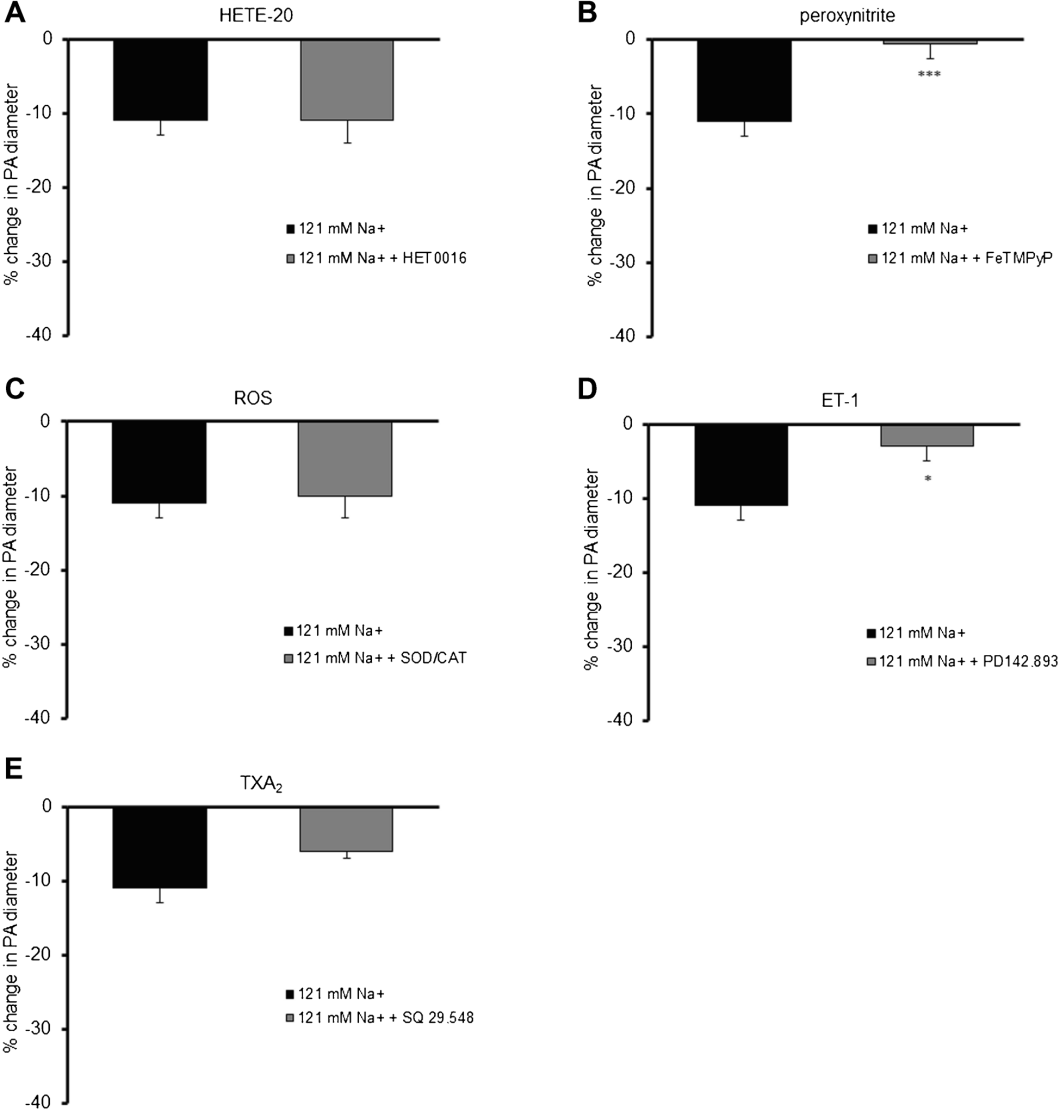
Mechanisms of constriction of parenchymal arterioles in low sodium environment (121 mM Na^+^, *n* = 9). **A** HET0016 (inhibitor of synthesis of 20-hydroxyeicosatetraenoic acid, 20-HETE, 10 µM, *n* = 5) did not influence constriction of PA; **B** Peroxynitrite decomposition catalyst (FeTMPyP, 10 µM, *n* = 5) abolished constriction of PA; **C** ROS scavengers: superoxide dismutase (SOD; 200 U/mL) and catalase (CAT, 150 U/mL, *n* = 4) did not influence constriction of PA; **D** Endothelin ET_A_/ET_B_ receptors antagonist (PD142,893, 100 nM, *n* = 5) abolished constriction of PA; **E** Thromboxane A_2_/prostaglandin H_2_ (PGH_2_) receptor blocker (SQ 29,548, 2 µM, *n* = 5) did not influence constriction of PA observed in low sodium environment. The change in PA diameter was calculated as a percentage of the baseline diameter. Data are expressed as means ± S.E.M., *p < 0.05, ***p < 0.001 vs. 121 mM Na^+^ conditions

Since the decrease in sodium ion concentration in the intra- and extravascular buffer without the presence of vasopressin also induced slight, but statistically significant constriction of parenchymal arterioles (by 11 ± 2%, *p* < 0.01, *n* = 9), the mechanism of this vasospasm was also assessed. Constriction of parenchymal arterioles in low sodium conditions was abolished by peroxynitrite decomposition catalyst (FeTMPyP, 10 µM, *n* = 5) (Fig. 6C), and endothelin ET_A_/ET_B_ receptors antagonist (PD142,893, 100 nM, *n* = 5) (Fig. 6E), but in contrast to the low sodium/AVP conditions, SOD/CAT did not affect constriction of parenchymal arterioles (Fig. 6D).

### Mechanism of the disturbed response of parenchymal arterioles to ATP in low sodium environment in the presence of vasopressin

**Fig. 7:**
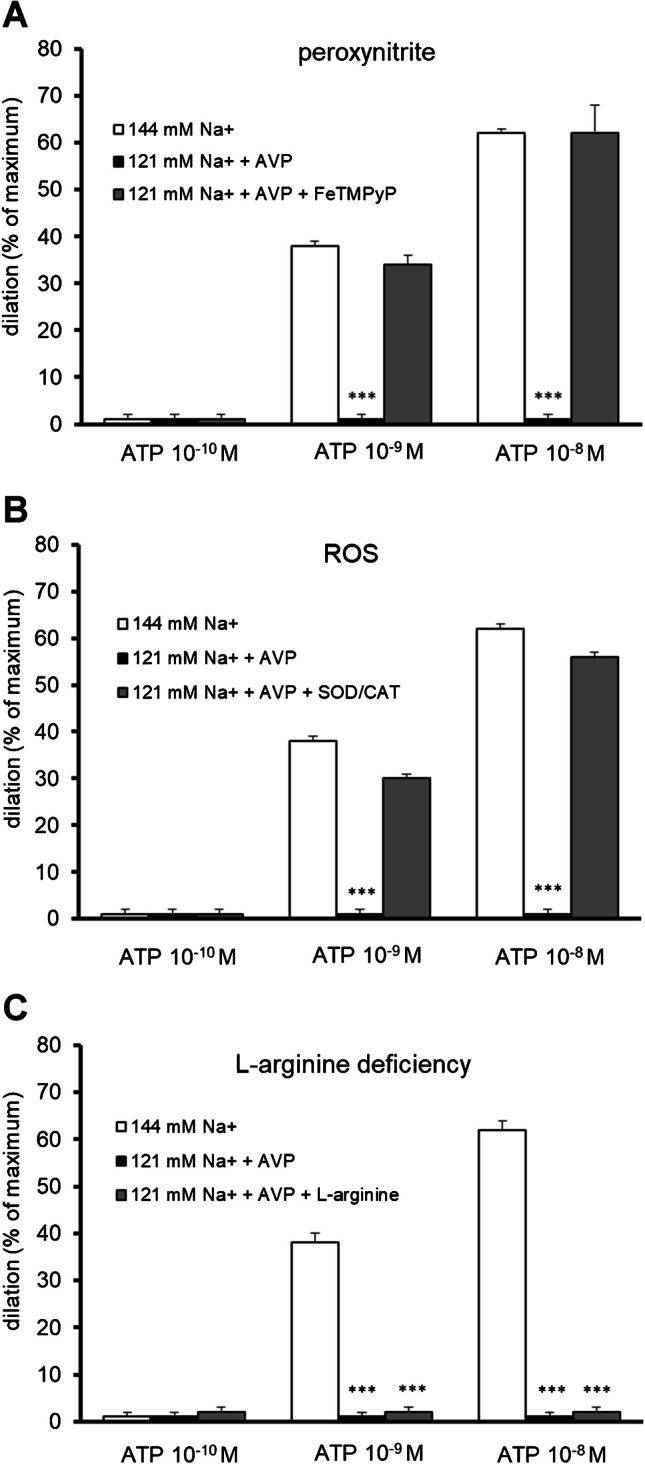
Mechanisms of the disturbed response of parenchymal arterioles to ATP (100 pM–10 nM) in low sodium environment (121 mM Na^+^) in the presence of vasopressin (15 pg/ml). **A** Peroxynitrite decomposition catalyst (FeTMPyP, 10 µM, *n* = 5) restored the dilation of PA to ATP, **B** ROS scavengers: superoxide dismutase (SOD; 200 U/mL) and catalase (CAT, 150 U/mL, *n* = 5) restored the dilation of PA to ATP;** C** NO precursor, L-arginine (1 nM, *n* = 6), did not improve the disturbed response of PA to ATP. The dilation is expressed as a percentage of the maximum diameter (0 Ca^2+^, EGTA 1 mM). Data are expressed as means ± S.E.M., ***p < 0.001 vs. physiological conditions (i.e., 144 mM Na^+^ concentration)

The mechanisms underlying NO depletion can be broadly divided into processes that transform bioavailable NO into other species (e.g., peroxynitrite) and deficiencies in the production of NO [13]. The present study demonstrated that both peroxynitrite decomposition catalyst (FeTMPyP, 10 µM, *n* = 5), and ROS scavengers: superoxide dismutase (SOD; 200 U/mL) and catalase (CAT, 150 U/mL, *n* = 5) completely restored dilation of the parenchymal arterioles to ATP 1 nM (vasodilation by 32 ± 2%, *p* < 0.001, and by 30 ± 1%, *p* < 0.001, respectively), and to ATP 10 nM vasodilation by 62 ± 6%, *p* < 0.001, and by 56 ± 3%, *p* < 0.001, respectively) in low sodium environment in the presence of vasopressin, (Fig. 7A, B). In contrast, the precursor of NO, L-arginine (1 nM, *n* = 6), did not influence the disturbed response of parenchymal arterioles to ATP; that is, no dilation was observed (Fig. 7C). The relatively low dose of L-arginine (1 nM) used in these experiments was dictated by pilot studies that had shown that higher doses of L-arginine (10 nM–10 µM) induced maximal dilation of parenchymal arterioles, completely exhausting the vasodilatory reserve. Therefore, it was not feasible to test whether ATP in the background of L-arginine leads to dilation of parenchymal arterioles. L-arginine, at a dose of 1 nM, caused vasodilation while still preserving the vasodilatory reserve.

### Superoxide formation in parenchymal arterioles in low sodium environment in the presence of vasopressin

**Fig. 8:**
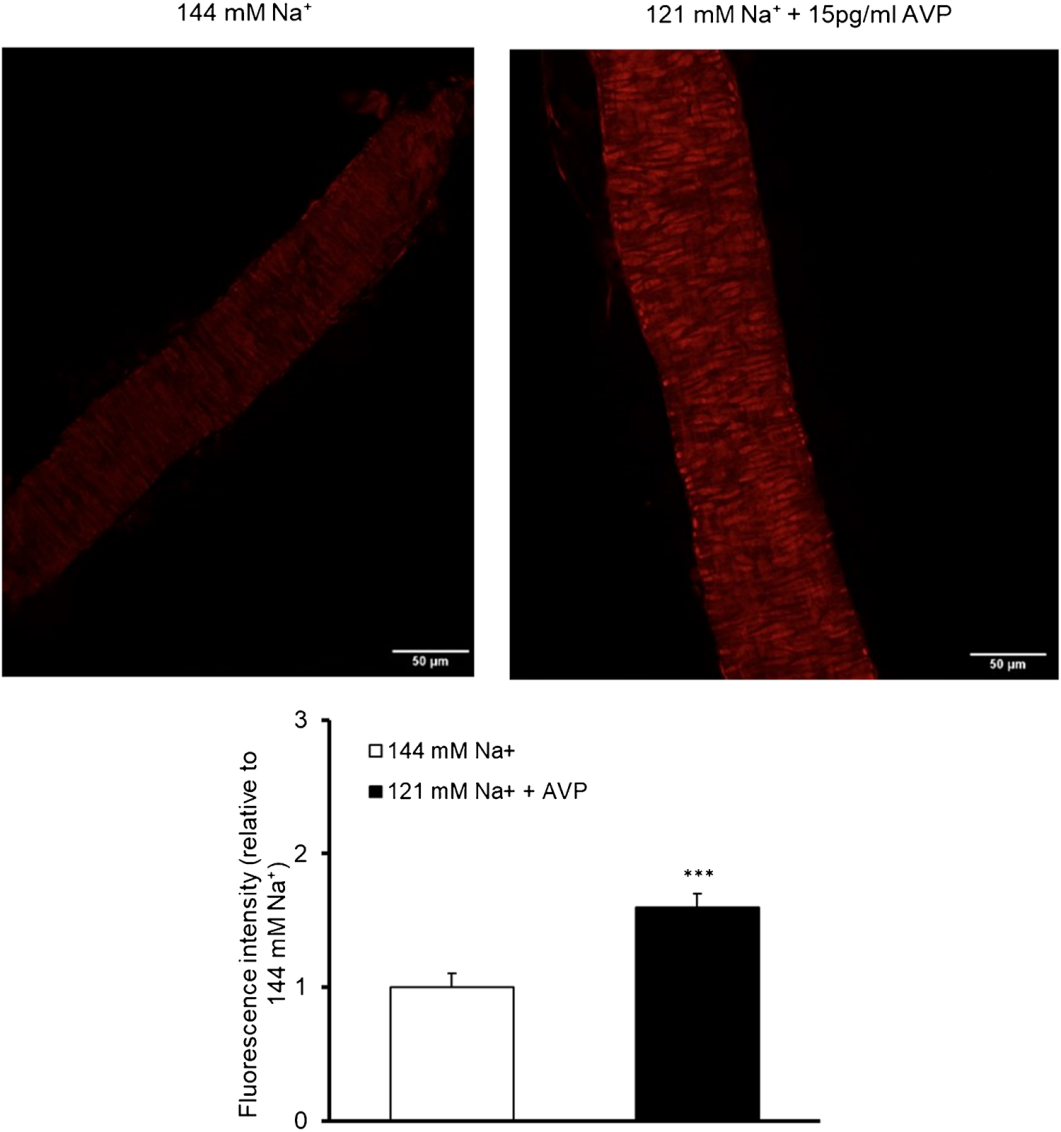
Superoxide formation, assessed by the dihydroethidium (DHE) staining, in isolated rat’s parenchymal arterioles in physiological conditions (i.e., 144 mM Na^+^, *n* = 4) and in low sodium environment (121 mM Na^+^) in the presence of vasopressin (15 pg/ml) (*n* = 5). 482 regions were analyzed in total. Data are presented as mean ± S.E.M., normalized to physiological conditions, ***p < 0.001 vs. physiological conditions (i.e., 144 mM Na^+^ concentration). Scale bar = 50 µm

Incubation of isolated parenchymal arterioles in the increased concentration of AVP to 15 pg/ml and decreased concentration of Na^+^ to 121 mM elevated the fluorescence signal of ethidium in the nuclei of vascular cells (Fig. 8). These data indicated that in low sodium environment in the presence of vasopressin, superoxide production in cerebral parenchymal arterioles was greater compared to control conditions.

## Discussion

All studies presented in this article were performed on isolated rat’s intracerebral small vessels − parenchymal arterioles in low sodium environment in the presence of vasopressin, which mimics AVP-associated hyponatremia in vivo. The purpose of the present study was to investigate the mechanism of dysfunction, that is, constriction and impaired endothelial regulation of parenchymal arterioles in conditions of decreased Na^+^ concentration to 121 mM and increased AVP concentration to 15 pg/ml. The results of the present study demonstrated that the dysfunction of small vessels in these conditions is associated with (1) decreased involvement of NO in maintaining the basal tone of parenchymal arterioles, (2) involvement of oxidative/nitrosative stress, activation of V_1a_ and ET_A_/ET_B_ receptors. Metabolites of arachidonic acid, such as CYP450 4 A-derived 20-HETE and COX-derived TXA_2,_ were not involved in the studied mechanisms. Administration of NO precursor, L-arginine, likewise did not improve vascular disorders observed in low sodium environment in the presence of vasopressin. Moreover, the present studies revealed increased superoxide formation in such conditions.

The combination of low sodium and vasopressin that mimic hyponatremia was selected for the present studies since previous results showed that the mere reduction of sodium ion concentration led only to a slight constriction of parenchymal arterioles without affecting endothelial regulation [2, 3]. These observations indicated that low sodium leads to a lesser dysfunction of parenchymal arterioles compared to the simultaneous effects of low sodium and AVP. Regardless of whether the cause of hyponatremia is an inappropriate secretion of antidiuretic hormone or cerebral salt wasting syndrome, patients usually present with elevated plasma concentrations of vasopressin [1, 25, 33, 40, 41].

Previous studies have shown that in low sodium conditions in the presence of vasopressin, the parenchymal arterioles demonstrated an impaired response to ATP [2, 3], an observation indicative of endothelial dysfunction. It was demonstrated that small doses of ATP cause cerebral vessel dilation through a mechanism involving nitric oxide (NO) production [27]. Current data demonstrate that in low sodium environment in the presence of vasopressin, the role of NO in maintaining basal tone is significantly reduced, likely due to NO depletion. Consequently, under these conditions, both basal and ATP-stimulated secretion of nitric oxide was diminished. Interestingly, however, the correction of osmolality to physiological level, with maintaining low sodium conditions and the presence of vasopressin restored arteriolar dilation in response to ATP, but did not affect the vasoconstriction. The leading causes of reduced NO-dependent responses are either decreased production or increased inactivation of NO [13]. The common reason for both processes is oxidative stress and excessive superoxide anion production (O2^•−^). The superoxide anion binds nitric oxide in a reaction that produces peroxynitrite (ONOO^−^), consequently reducing NO bioavailability [13, 15]. Treatment of purified eNOS with peroxynitrite significantly decreased the ability of the enzyme to produce NO [37]. In turn, uncoupled eNOS produced superoxide anion instead of NO, further increasing vascular superoxide production [32]. The present studies showed that this common mechanism of NO inactivation by oxidative/nitrosative stress is the cause of dysfunction of parenchymal arterioles in low sodium environment in the presence of vasopressin. Both peroxynitrite decomposition catalyst and ROS scavengers diminished the constriction and restored the response of parenchymal arterioles to ATP. Dihydroethidium staining confirmed increased superoxide formation in parenchymal arterioles in these conditions.

Besides the increased inactivation of nitric oxide, the observed depletion of NO could also likely be due to a deficiency of its precursor, L-arginine [13]. Current studies showed that adding L-arginine to the buffer did not improve the response of parenchymal arterioles to ATP; thus, the impaired ATP response was unrelated to L-arginine deficiency. Other studies evaluating the effect of L-arginine on improving NO-dependent responses are inconsistent. For example, Faraco et al. [17] showed that treatment with L-arginine abolished the vascular NO deficits observed in a high salt diet. On the other hand, other studies showed no effect of L-arginine administration on endothelial dysfunction [4, 47]. Some data indicate that longer-term exposure to L-arginine can be detrimental to vascular function due to suppressing eNOS activity and intensifying oxidative stress [34]. The lack of improved responses of parenchymal arterioles to ATP may be associated with the ongoing degradation of nitric oxide by oxidative and nitrosative stress.

Forward analysis implies the need to understand better the mechanism responsible for generating oxidative stress in low sodium environment in the presence of vasopressin. The production of ROS may be related to the presence of vasopressin in the buffer. Some data demonstrate that SR 49059, an inhibitor of the vasopressin V_1a_ receptor, improved the dehydration-induced oxidative stress [18]. In the present study, SR 49059 abolished constriction observed in low sodium environment in the presence of vasopressin, indicating vasopressin's involvement in the studied disorder. On the other hand, we and others [43, 44] demonstrated that low doses of AVP administered under physiological conditions led to dilation of parenchymal arterioles and this effect is also dependent on V_1a_ receptors, since SR 49059 abolished AVP-induced vasorelaxation. In low sodium conditions, the same dose of AVP caused vasoconstriction. Yet, the relaxation of the vessels by AVP was due to the secretion of nitric oxide [43, 44]. NO deficiency, resulting from the simultaneous action of AVP and low sodium, may be why AVP did not dilate as in physiological conditions. Low sodium also stimulates oxidative stress [8, 9]. However, an earlier study from our laboratory showed a lesser dysfunction of parenchymal arterioles in low sodium buffer compared to increased concentration of AVP in the presence of low sodium conditions [2, 3]. Moreover, the present studies showed that slight constriction of parenchymal arterioles in low sodium environment was dependent on endothelin ET_A/B_ receptors and peroxynitrite, but ROS scavengers did not affect this response. This is probably associated with ET-1_B_ receptor activations, which typically cause vasodilation, but in certain conditions could directly induce peroxynitrite that leads to constriction of cerebral arterioles [12]. In further support of these observations, Angiotensin II, another agent that, like vasopressin, led to endothelium-dependent vasodilation and induced oxidative stress [21], in combination with low sodium, did not induce vasoconstriction [1]. Altogether, the apparent constriction of parenchymal arterioles and induction of oxidative stress was unique to the combined action of both factors.

In association with NO deficiency, the endothelium can control vascular tone by producing vasoconstrictive substances such as endothelin-1, thromboxane A_2_, prostaglandin H_2,_ and 20-HETE [15, 28, 38]. The present study showed that constriction of parenchymal arterioles in low sodium environment in the presence of vasopressin was abolished by ET-1_A/B_ receptor antagonist but not by thromboxane A_2_/prostaglandin H_2_ receptor antagonist. Likewise, an inhibitor of biosynthesis of 20-HETE, HET0016, did not improve vasoconstriction. Thus, metabolites of arachidonic acid, such as CYP450 4 A-derived 20-HETE and COX-derived TXA_2_ are not involved in the studied dysfunction of parenchymal arterioles. Endothelin-1, in turn, contributed to constricting parenchymal arterioles in the presence of increased AVP concentration and low sodium. Another study showed that AVP may stimulate endothelin-1 secretion, but only after inhibition of NO synthase [38], i.e., conditions such as those observed in low sodium environment in the presence of vasopressin. Disturbed secretion of NO and impaired function of the endothelium of cerebral parenchymal arterioles are the basis for small blood vessel diseases [14]. Endothelial dysfunction may lead to cognitive impairment by reducing resting cerebral blood flow. Nitric oxide and its vascular effects are also potent neuromodulators required for memory formation [29]. Moreover, experimental studies using small blood vessels revealed that oxidative stress might be a common mechanism responsible for cerebral vascular dysfunction [14].

In summary, the present studies showed that increased vasopressin concentration in the presence of decreased Na^+^ concentration leads to dysfunction of small intracerebral arterioles via oxidative/nitrosative stress. The leading recognized cause of hyponatremia symptoms, such as reduced consciousness, confusion, headache, and attention deficits, is hypoosmotic brain edema [7, 25, 40, 42]. The presence of cognitive impairment in the experimental model of chronic hyponatremia [18, 29], despite fully developed adaptive mechanisms and lack of brain edema [7, 46], indicates an additional cause of observed dysfunction. Although the present studies were performed in vitro, they shed new light on the correlation between dysfunction of small blood vessels and cognitive deficits [19, 30].

## Data Availability

The datasets generated and analysed during the current study are available at the RepOD repository 10.18150/I7MLWB.

## References

[1] Aleksandrowicz M, Klapczynska K, Kozniewska E (2020) Dysfunction of the endothelium and constriction of the isolated rat’s middle cerebral artery in low sodium environment in the presence of vasopressin. Clin Exp Pharmacol Physiol 47(5):759–764. 10.1111/1440-1681.13242 31876005 10.1111/1440-1681.13242

[2] Aleksandrowicz M, Kozniewska E (2020) Compromised regulation of the rat brain parenchymal arterioles in vasopressin-associated acute hyponatremia. Microcirculation 27(7):e12644. 10.1111/micc.12644 32603523 10.1111/micc.12644

[3] Aleksandrowicz M, Kozniewska E (2022) Hyponatremia as a risk factor for microvascular spasm following subarachnoid hemorrhage. Exp Neurol 355:114126. 10.1016/j.expneurol.2022.114126 35654161 10.1016/j.expneurol.2022.114126

[4] Alvares TS, Conte-Junior CA, Silva JT, Paschoalin VM (2014) L-Arginine does not improve biochemical and hormonal response in trained runners after 4 weeks of supplementation. Nutr Res 34(1):31–39. 10.1016/j.nutres.2013.10.006 24418244 10.1016/j.nutres.2013.10.006

[5] Arieff AI, Ayus JC (1993) Pathogenesis of hyponatremic encephalopathy. Curr Concepts Chest 103(2):607–610. 10.1378/chest.103.2.607 10.1378/chest.103.2.607 8432163

[6] Arieff AI, Guisado R (1997) Effects on the central nervous system of hypernatremic and hyponatremic states. Kidney Int 10(1):104–116. 10.1038/ki.1976.82 10.1038/ki.1976.82 7702

[7] Ayus JC, Achinger SG, Arieff A (2008) Brain cell volume regulation in hyponatremia: role of sex, age, vasopressin, and hypoxia. Am J Physiol Renal Physiol 295:619–624. 10.1152/ajprenal.00502.2007 10.1152/ajprenal.00502.2007 18448591

[8] Barsony J, Sugimura Y, Verbalis JG (2011) Osteoclast response to low extracellular sodium and the mechanism of hyponatremia-induced bone loss. JBC 286:10864–10875 10.1074/jbc.M110.155002 PMC3060537 21135109

[9] Benvenuti S, Deledda C, Luciani P, Modi G, Bossio A, Giuliani C, Fibbi B, Peri A (2013) Low extracellular sodium causes neuronal distress independently of reduced osmolality in an experimental model of chronic hyponatremia. Neuromolecular Med 15(3):493–503. 10.1007/s12017-013-8235-0 23695860 10.1007/s12017-013-8235-0

[10] Berl T (2013) An elderly patient with chronic hyponatremia. Clin J Am Soc Nephrol 8:469–475. 10.2215/CJN.03100312 23037983 10.2215/CJN.03100312

[11] Cassuto J, Dou H, Czikora I, Szabo A, Patel VS, Kamath V, Belin de Chantemele E, Feher A, Romero MJ, Bagi Z (2014) Peroxynitrite disrupts endothelial caveolae leading to eNOS uncoupling and diminished flow-mediated dilation in coronary arterioles of diabetic patients. Diabetes 63:1381–1393. 10.2337/db13-0577 24353182 10.2337/db13-0577 PMC3964507

[12] Cipolla MJ, Sweet JG, Gokina NI, White SL, Nelson MT (2013) Mechanisms of enhanced basal tone of brain parenchymal arterioles during early postischemic reperfusion: role of ET-1-induced peroxynitrite generation. J Cereb Blood Flow Metab 33(10):1486–1492. 10.1038/jcbfm.2013.99 23778163 10.1038/jcbfm.2013.99 PMC3790940

[13] Cyr AR, Huckaby LV, Shiva SS, Zuckerbraun BS (2020) Nitric oxide and endothelial dysfunction. Crit Care Clin 36(2):307–321. 10.1016/j.ccc.2019.12.009 32172815 10.1016/j.ccc.2019.12.009 PMC9015729

[14] De Silva TM, Miller AA (2016) Cerebral small vessel disease: targeting oxidative stress as a novel therapeutic strategy? Front Pharmacol 7:61. 10.3389/fphar.2016.00061 27014073 10.3389/fphar.2016.00061 PMC4794483

[15] Faraci FM (2006) Reactive oxygen species: influence on cerebral vascular tone. J Appl Physiol 100:739–743. 10.1152/japplphysiol.01044.2005 16421281 10.1152/japplphysiol.01044.2005

[16] Faraci FM, Brian JE Jr (1994) Nitric oxide and the cerebral circulation. Stroke 25(3):692–703. 10.1161/01.str.25.3.692 7510430 10.1161/01.str.25.3.692

[17] Faraco G, Brea D, Garcia-Bonilla L, Wang G, Racchumi G, Chang H, Buendia I, Santisteban MM, Segarra SG, Koizumi K, Sugiyama Y, Murphy M, Voss H, Anrather J, Iadecola C (2018) Dietary salt promotes neurovascular and cognitive dysfunction through a gut-initiated TH17 response. Nat Neurosci 21(2):240–249. 10.1038/s41593-017-0059 29335605 10.1038/s41593-017-0059-z PMC6207376

[18] Faraco G, Wijasa TS, Park L, Moore J, Anrather J, Iadecola C (2014) Water deprivation induces neurovascular and cognitive dysfunction through vasopressin-induced oxidative stress. J Cereb Blood Flow Metab 34(5):852–860. 10.1038/jcbfm.2014.24 24517977 10.1038/jcbfm.2014.24 PMC4013763

[19] Fujisawa H, Sugimura Y, Takagi H, Mizoguchi H, Takeuchi H, Izumida H, Nakashima K, Ochiai H, Takeuchi S, Kiyota A, Fukumoto K, Iwama S, Takagishi Y, Hayashi Y, Arima H, Komatsu Y, Murata Y, Oiso Y (2016) Chronic hyponatremia causes neurologic and psychologic impairments. J Am Soc Nephrol 27:766–780. 10.1681/ASN.2014121196 26376860 10.1681/ASN.2014121196 PMC4769197

[20] Gimbrone MA, García-Cardeña G (2016) Endothelial cell dysfunction and the pathobiology of atherosclerosis. Circ Res 118:620–636. 10.1161/CIRCRESAHA.115.306301 26892962 10.1161/CIRCRESAHA.115.306301 PMC4762052

[21] Girouard H, Park L, Anrather J, Zhou P, Iadecola C (2007) Cerebrovascular nitrosative stress mediates neurovascular and endothelial dysfunction induced by angiotensin II. Arterioscler Thromb Vasc Biol 27:303–309. 10.1161/01.ATV.0000253885.41509.25 17138940 10.1161/01.ATV.0000253885.41509.25

[22] Gorelick PB, Pantoni L (2013) Advances in vascular cognitive impairment. Stroke 44(2):307–308. 10.1161/STROKEAHA.111.000219 23321448 10.1161/STROKEAHA.111.000219

[23] Gunathilake R, Oldmeadow C, McEvoyM KB, Inder K, Schofield P, Attia J (2013) Mild hyponatremia is associated with impaired cognition and falls in community-dwelling older persons. J Am Geriatr Soc 61:1838–1839. 10.1111/jgs.12468 24117308 10.1111/jgs.12468

[24] Hadi HAR, Carr CS, Al Suwaidi J (2005) Endothelial dysfunction: cardiovascular risk factors, therapy, and outcome. Vasc Health Risk Manag 1:183–198 17319104 PMC1993955

[25] Hannon MJ, Behan LA, O’Brien MM, Tormey W, Ball SG, Javadpour M, Sherlock M, Thompson CJ (2014) Hyponatremia following mild/moderate subarachnoid hemorrhage is due to SIAD and glucocorticoid deficiency and not cerebral salt wasting. J Clin Endocrinol Metab 99:291–298. 10.1210/jc.2013-3032 24248182 10.1210/jc.2013-3032

[26] Hannon MJ, Thompson CJ (2014) Neurosurgical hyponatremia. J Clin Med 3:1084–1104 26237593 10.3390/jcm3041084 PMC4470172

[27] Horiuchi T, Dietrich HH, Hongo K, Dacey RG Jr (2003) Comparison of P2 receptor subtypes producing dilation in rat intracerebral arterioles. Stroke 34(6):1473–1478. 10.1161/01.STR.0000071527.10129.65 12730558 10.1161/01.STR.0000071527.10129.65

[28] Imig JD, Simpkins AN, Renic M, Harder DR (2011) Cytochrome P450 eicosanoids and cerebral vascular function. Expert Rev Mol Med 13:e7. 10.1017/S1462399411001773 21356152 10.1017/S1462399411001773 PMC3613250

[29] Katusic ZS, Austin SA. Eur Heart J (2014) Endothelial nitric oxide: protector of a healthy mind. 35(14):888–894. 10.1093/eurheartj/eht544. 10.1093/eurheartj/eht544 PMC3977136 24357508

[30] Kawakami T, Fujisawa H, Nakayama S, Yoshino Y, Hattori S, Seino Y, Takayanagi T, Miyakawa T, Suzuki A, Sugimura Y (2021) Vasopressin escape and memory impairment in a model of chronic syndrome of inappropriate secretion of antidiuretic hormone in mice. Endocr J 68(1):31–43. 10.1507/endocrj.EJ20-0289 32879162 10.1507/endocrj.EJ20-0289

[31] Li Q, Yang Y, Reis C, Tao T, Li W, Li X, Zhang JH (2018) Cerebral small vessel disease. Cell Transplant 27(12):1711–1722. 10.1177/0963689718795148 30251566 10.1177/0963689718795148 PMC6300773

[32] Łuczak A, Madej M, Kasprzyk A, Doroszko A (2020) Role of the eNOS Uncoupling and the nitric oxide metabolic pathway in the pathogenesis of autoimmune rheumatic diseases. Oxid Med Cell Longev 1417981. 10.1155/2020/1417981 10.1155/2020/1417981 PMC7174952 32351667

[33] Mather HM, Ang V, Jenkins JS (1981) Vasopressin in plasma and CSF of patients with subarachnoid haemorrhage. J Neurol Neurosurg Psychiatry 44:216–219. 10.1136/jnnp.44.3.216 7229644 10.1136/jnnp.44.3.216 PMC490894

[34] Mohan S, Wu CC, Shin S, Fung HL (2012) Continuous exposure to L-arginine induces oxidative stress and physiological tolerance in cultured human endothelial cells. Amino Acids 43(3):1179–1188. 10.1007/s00726-011-1173-y 22130739 10.1007/s00726-011-1173-y PMC3321093

[35] Nakagomi T, Kassell NF, Sasaki T, Fujiwara S, Lehman RM, Torner JC (1987) Impairment of endothelium-dependent vasodilation induced by acetylcholine and adenosine triphosphate following experimental subarachnoid hemorrhage. Stroke 18:482–489. 10.1161/01.str.18.2.482 3564107 10.1161/01.str.18.2.482

[36] Olson JE, Mishler L, Dimlich RV (1990) Brain water content, brain blood volume, blood chemistry, and pathology in a model of cerebral edema. Ann Emerg Med 19(10):1113–1121. 10.1016/s0196-0644(05)81514-8 2221516 10.1016/s0196-0644(05)81514-8

[37] Pasquet JP, Zou MH, Ullrich V (1996) Peroxynitrite inhibition of nitric oxide synthases. Biochimie 78(8–9):785–791. 10.1016/s0300-9084(97)82537-0 9010608 10.1016/s0300-9084(97)82537-0

[38] Postma CT, Maessen SMJ, Thien T, Smits P (2005) The effect of arginine vasopressin on endothelin production in the human forearm vascular bed. Neth J Med 63:199–204 16011011

[39] Quick S, Moss J, Rajani RM, Trends WA, Neurosci, (2021) A vessel for change: Endothelial dysfunction in cerebral small vessel disease. Trends Neurosci 44(4):289–305. 10.1016/j.tins.2020.11.003 33308877 10.1016/j.tins.2020.11.003

[40] Rabinstein AA, Wijdicks EFM (2003) Hyponatremia in critically Ill neurological patients. Neurologist 9:290–300. 10.1097/01.nrl.0000095258.07720.89 14629783 10.1097/01.nrl.0000095258.07720.89

[41] Sherlock M, O’Sullivan E, Agha A, Behan LA, Rawluk D, Brennan P, Tormey W, Thompson CJ (2006) The incidence and pathophysiology of hyponatraemia after subarachnoid haemorrhage. Clin Endocrinol (Oxf) 64(3):250–254. 10.1111/j.1365-2265.2006.02432.x 16487432 10.1111/j.1365-2265.2006.02432.x

[42] Sterns RH (2018) Treatment of severe hyponatremia. Clin J Am Soc Nephrol 13(4):641–649. 10.2215/CJN.10440917 29295830 10.2215/CJN.10440917 PMC5968908

[43] Suzuki Y, Satoh S, Oyama H, Takayasu M, Shibuya M (1993) Regional differences in the vasodilator response to vasopressin in canine cerebral arteries in vivo. Stroke 24:1049–1053 7686696 10.1161/01.str.24.7.1049

[44] Takayasu M, Kajita Y, Suzuki Y, Shibuya M, Sugita K, Ishikawa T, Hidaka H (1993) Triphasic response of rat intra cerebral arterioles to increasing concentrations of vasopressin in vitro. J Cereb Blood Flow Metab 13:304–309 8436623 10.1038/jcbfm.1993.38

[45] Toth J, Racz A, Kaminski PM, Wolin MS, Bagi Z, Koller A (2007) Asymmetrical dimethylarginine inhibits shear stress-induced nitric oxide release and dilation and elicits superoxide-mediated increase in arteriolar tone. Hypertension 49(3):563–568. 10.1161/01.HYP.0000256764.86208.3d 17242303 10.1161/01.HYP.0000256764.86208.3d

[46] Verbalis JG (1993) Hyponatremia induced by vasopressin or desmopressin in female and male rats. J Am Soc Nephrol 3(9):1600–1606. 10.1681/ASN.V391600 8507816 10.1681/ASN.V391600

[47] Walker HA, McGing E, Fisher I, Böger RH, Bode-Böger SM, Jackson G, Ritter JM, Chowienczyk PJ (2001) Endothelium-dependent vasodilation is independent of the plasma L-arginine/ADMA ratio in men with stable angina: lack of effect of oral L-arginine on endothelial function, oxidative stress, and exercise performance. J Am Coll Cardiol 38(2):499–505. 10.1016/S0735-1097(01)01380-8 11499744 10.1016/s0735-1097(01)01380-8

